# Negative Ions Enhance Survival of Membrane Protein Complexes

**DOI:** 10.1007/s13361-016-1381-5

**Published:** 2016-04-22

**Authors:** Idlir Liko, Jonathan T. S. Hopper, Timothy M. Allison, Justin L. P. Benesch, Carol V. Robinson

**Affiliations:** Department of Chemistry, University of Oxford, South Parks Road, Oxford, OX1 5QY UK

**Keywords:** Membrane proteins, Negative polarity, Structural biology

## Abstract

Membrane protein complexes are commonly introduced to the mass spectrometer solubilized in detergent micelles. The collisional activation used to remove the detergent, however, often causes protein unfolding and dissociation. As in the case for soluble proteins, electrospray in the positive ion mode is most commonly used for the study of membrane proteins. Here we show several distinct advantages of employing the negative ion mode. Negative polarity can yield lower average charge states for membrane proteins solubilized in saccharide detergents, with enhanced peak resolution and reduced adduct formation. Most importantly, we demonstrate that negative ion mode electrospray ionization (ESI) minimizes subunit dissociation in the gas phase, allowing access to biologically relevant oligomeric states. Together, these properties mean that intact membrane protein ions can be generated in a greater range of solubilizing detergents. The formation of negative ions, therefore, greatly expands the possibilities of using mass spectrometry on this intractable class of protein.

**Electronic supplementary material:**

The online version of this article (doi:10.1007/s13361-016-1381-5) contains supplementary material, which is available to authorized users.

**Graphical Abstract:**
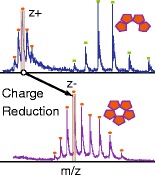
ᅟ

## Introduction

Mass spectrometry (MS) of intact membrane proteins and their complexes has developed rapidly over the past 10 years [1]. Knowledge of protein isolation and preparation protocols are sufficiently established but challenges remain, including the insolubility and instability of many membrane proteins in the detergents preferred for MS. Consequently, new approaches are needed to provide opportunity for MS studies of a greater range of membrane proteins.

Following solubilization in detergents, membrane proteins are introduced to the mass spectrometer embedded in micelles. Subsequently, the detergent is removed in the gas phase, allowing the membrane proteins to be accurately mass measured [2, 3]. Although the presence of detergent allows the membrane proteins to maintain folded conformations in solution, the requirement to remove the detergent, by collisional activation, often causes dissociation of protein subunits. Factors that influence the preservation of complexes in a MS experiment include: (1) the time ions spend in the gas phase, (2) the charge state of the ions, and (3) the acceleration voltage. However, the timescales required for major structural rearrangements are notably longer [4, 5] than a typical MS measurement. The amount of collisional activation, however, which in itself is a function of charge state, plays an important role in maintaining membrane proteins in ‘native-like’ states [6].

As detergent is removed by collisional activation, resolved spectra are typically achieved at elevated energies, relative to those typically employed for soluble proteins. This can hamper the ability to maintain the integrity of membrane protein structure. To lower the effect of collision-induced unfolding and dissociation, detergents that can be removed at the lowest collisional energies are favored. Previously we have shown that the detergents lauryldimethylamine *N*-oxide (LDAO) and tetraethylene glycol monooctyl ether (C8E4) can be removed at significantly lower energies [6], which better preserves the oligomeric states, and also the folded conformations of membrane proteins. In addition, as well as effectively being ‘volatile’ detergents, both facilitate the production of ions with charge states lower than would otherwise be expected. Consequently, the extent of Coulombic unfolding and dissociation of membrane proteins is minimized. Both characteristics improve the ability to preserve the native oligomeric states and folded conformations of membrane proteins.

LDAO and C8E4 are classified as ‘harsh’ detergents in solution, which can lead to an enhanced degree of delipidation [7]. Frequently, therefore, these detergents destabilize the membrane protein in solution. ‘Mild’ detergents, such as the saccharide-based ones, require elevated energies to remove them in the gas phase. Furthermore, they do not possess charge reducing abilities, meaning that ions with higher charge states are observed. To counteract this, and lower the kinetic energy and degree of Coulombic repulsion, the strategy of recording mass spectra in the presence of charge-reducing agents was exploited [8]. Addition of imidazole to the solution, or acetonitrile in the atmosphere of the electrospray plume, reduces the average charge state of membrane protein ions. The presence of additives is not always desirable, however, and does not surmount the problem of the high collisional activation necessary to strip detergent.

Previous studies have shown that in negative polarity, the charge state distributions of soluble proteins are generally lower than when using positive polarity [9, 10]. By performing ion mobility measurements in both polarities, it was found that the experimentally determined collision cross sections were similar for the same charge states [11], and for the lower charge states recorded in negative polarity [12]. As the surface area of the proteins therefore remained the same, regardless of polarity, it was reasoned that the charged residue model (CRM), which depends on this factor, cannot, on its own, explain the observed charge state differences. Furthermore, as the proteins were introduced from identical conditions in both polarities, the observed differences in the charge states of the ions were attributed to carrier effects in accordance with the charged residue-field emission model [13]. It was hypothesized that anions have lower charge-carrier energy than the corresponding cation in negative and positive polarities respectively.

In light of these measurements, performed in negative polarity for soluble proteins, we investigated if the same phenomena are manifested in the presence of detergents for membrane proteins. Here we show that charge reduction in negative polarity is observed only for saccharide detergents, providing evidence that the charge residue-field emission model applies in the presence of detergents in the negative ion mode. Furthermore, negative polarity provides a convenient method of preserving the oligomeric states without the need to solubilize in alternate detergents or manipulating the membrane protein solutions with additives such as imidazole.

## Materials and Methods

Membrane proteins were expressed and purified in-house from bacterial cell lines as previously reported [6, 14, 15]. Proteins were isolated from the bacterial membrane, except for the voltage-dependent anion-selective channel (VDAC) that was expressed in inclusion bodies. Proteins were then extracted from the membrane with a high percentage concentration of detergent (Affymetrix, Santa Clara, CA, USA) as used previously [6]; OmpF was extracted with 10% OG and VDAC was refolded in 2% LDAO. Subsequently, immobilized metal ion affinity chromatography (IMAC) and size exclusion chromatography were employed to purify the proteins and remove lipid adducts. In all purification steps, the detergent concentration was maintained at twice the critical micelle concentration (CMC).

Detergent exchange was performed as previously reported [3] using a Superdex 200 5/150 (GE Healthcare Life Sciences, Little Chalfont, UK) size exclusion column. Prior to MS analysis proteins were concentrated to 10–15 μM and buffer-exchanged into 200 mM ammonium acetate (Sigma, St. Louis, MO, USA) with twice the CMC of the desired detergent. Charge reduction measurements were performed by supplementing the protein solution with 5 mM imidazole (Acros Organics, Morris Plains, NJ, USA), or exposure to acetonitrile (Sigma) vapors.

MS measurements were performed on a Synapt G1 (Waters, Milford, MA, USA) instrument optimized for high mass measurements. Proteins were ionized from borosilicate glass capillaries that were pulled to a smaller diameter and gold coated in-house [16]. Strictly, the same capillary was maintained for both positive and negative polarity measurements to minimize capillary and solution variability. The capillary and cone voltages were 1.8 kV and 200 V for positive mode and 1.1 kV and 140 V for negative polarity. For saccharide detergents, 180–200 V collisional voltage and argon gas flow rate at 3–4 mL/min, resulting in a pressure of 3.5 × 10^−2^ mbar, were applied to the trap to remove bound detergent from the proteins. For proteins solubilized in C8E4 and LDAO detergents, the applied voltage was 140–160 V. Other key instrumental parameters were 4–6 mbar source pressure, and 50 V transfer collision energy.

The average charge state distribution was determined by fitting a Gaussian distribution to the measured charge state distribution employing the PULSAR software [17].

## Results and Discussion

### Negative Polarity Promotes Lower Charge States in Saccharide Detergents

**Figure 1:**
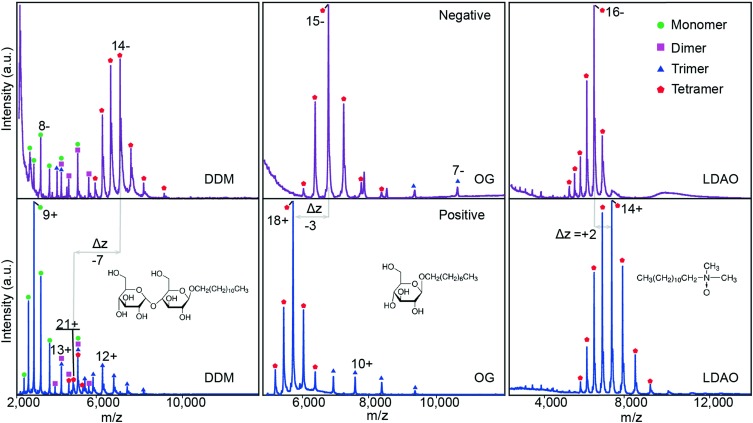
The effect of polarity on charge state distributions is dependent on detergent class. Mass spectra of AqpZ solubilized in DDM, OG, and LDAO (left, middle, and right panels, respectively) in negative polarity (top, purple) and positive polarity (bottom, blue). The oligomeric states for each case have been indicated. Inset show the structures of all three detergents

**Figure 2:**
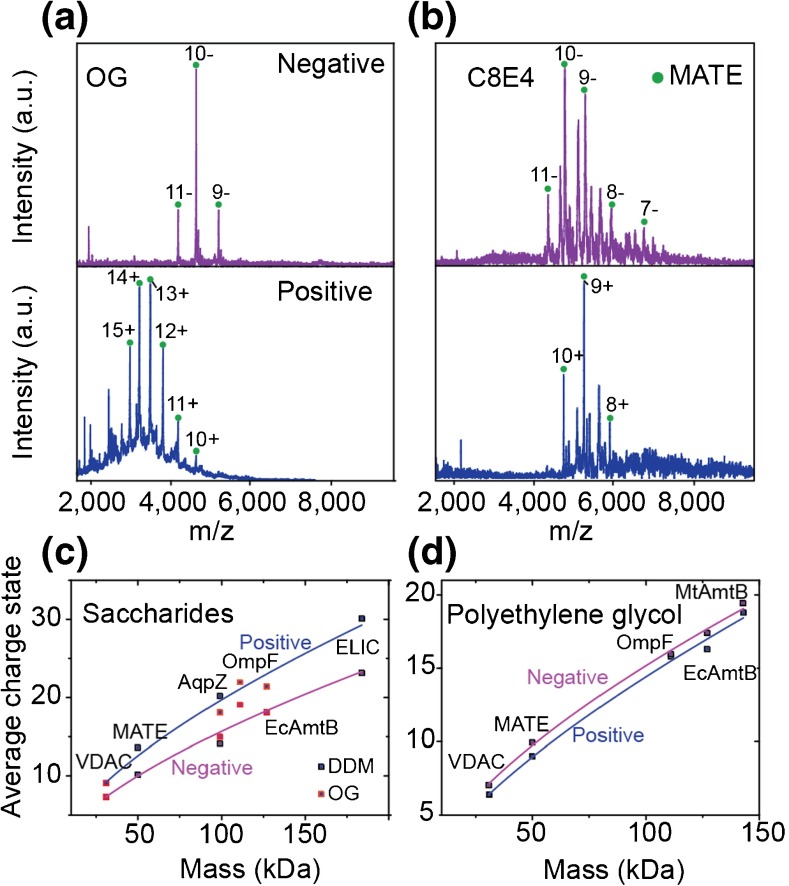
The effect of negative polarity on charge state in the presence of detergent. Mass spectra of MATE solubilized in OG (**a**) and C8E4 (**b**), in negative polarity (top, purple), and positive polarity (bottom, blue). A charge reduction was observed in the case of OG (left panel), whereas no charge reduction was observed for C8E4 (right panel). For the proteins investigated (Table S1) in saccharide detergents (**c**), a charge reduction is observed between positive (blue) and negative polarity (purple). The data are fitted to a power low (*z*_*ave*_ *= aM* ^*b*^), where *z*_*ave*_ is the average charge and *M* is mass in kDa, with *a* and *b* values of 0.99 and 0.65 for positive polarity and 0.78 and 0.65 for negative polarity. The *R* ^2^ of the fits were 0.97 and 0.95 for positive and negative ion modes respectively. (**d**) The dependence of average charge state on mass in polyethylene glycol detergents (C8E4) in both positive and negative polarity. The data were fitted with a power law with *a* and *b* values of 0.80 and 0.64 for negative mode and 0.61 and 0.69 for positive mode. The *R* ^2^ of both fits was 0.99

To investigate the dependence of charge-state distribution on polarity in the presence of detergent, mass spectra were recorded for three membrane proteins solubilized in saccharide detergents [n-dodecyl-β-D-maltopyranoside (DDM) or n-octyl-β-D-glucopyranoside (OG)], LDAO, and C8E4 (a polyethylene glycol detergent). First we recorded mass spectra for the water channel AqpZ, a tetrameric membrane protein, in DDM, OG, and LDAO in both positive and negative polarity (Figure 1). In positive polarity, the charge-state distributions were centered on 20.2+ and 18.1+ for the saccharide detergents DDM and OG, respectively. Maintaining the same solution conditions but recording the spectrum in negative polarity shifted the charge state distribution centers to 14.5– and 15.1–. This reduction in the charge state of approximately seven charges in DDM and three charges in OG stabilizes the tetrameric oligomeric state of the protein (Figure 1). Furthermore, measurements performed on AqpZ solubilized in LDAO showed no charge reduction but rather a charge increase between positive and negative polarity, from 14.5+ to 16.2–. For all other membrane proteins investigated here, in the saccharide detergents DDM or OG (Figures 1, 2, and Supplementary Figure S1) a charge reduction in negative polarity, relative to positive polarity, was observed.

We next considered membrane proteins solubilized in the polyethylene glycol detergent C8E4 (Figure 2b, and Supplementary Figure S1). Spectra of *Pyrococcus furiosus* multidrug and toxic compound extrusion protein (MATE) and the *E. coli* ammonia channel (AmtB), both solubilized in C8E4, show no significant difference in charge-state distributions between measurements made in positive and negative polarities; for MATE the average charge states were 9.0+ and 10.0–, and for *Ec*AmtB 16.3+ and 17.4– for positive and negative polarity, respectively. Spectra were then recorded for both proteins solubilized in OG. A reduction in the average charge state was observed from 21.4+ to 18.1– from positive to negative polarity for *Ec*AmtB (Supplementary Figure S1) and from 13.6+ to 9.8– for MATE (Figure 2). These measurements provide the first evidence that the detergent class determines the difference in charge states between polarities with saccharide detergents exhibiting the most marked effects.

To further examine the dependence of charge-state on polarity, we recorded mass spectra of seven additional membrane proteins, in a mass range from 31 to 184 kDa, in both polyethylene glycol and saccharide detergents. Notably, as protein mass increases, the average charge-state difference between positive and negative polarity increases when the membrane proteins are analyzed in the presence of saccharide detergents (Figure 2c and Supplementary Figure S2). A reduction in the difference of the charge-states is observed, however, for OG relative to DDM. More importantly, when spectra of the same proteins in C8E4 are recorded, there is no appreciable difference in average charge-state between the polarities (Figure 2d). In light of the combined charged residue-field emission model [13], this difference in average charge state, which depends on both polarity and detergent class, can be attributed to a charge-carrier effect, achieved by either acetate or polyethylene glycol molecules in negative and positive polarities, respectively, or a combination of charge being carried by both detergents and acetate. In negative polarity and with saccharide detergents, buffer particles leave with some charge, unlike their behavior in positive polarity. Analogous to the positive ion mode, polyethylene glycol detergents also leave with charge. Membrane proteins with greater mass presumably have greater surface area, and during the final desolvation steps of the electrospray process more buffer particles will leave carrying charge.

### Preserving the Native Oligomeric State by Reducing Coulombic Repulsion

For soluble proteins, negative polarity did not significantly improve preservation of native oligomeric states [12]. For membrane proteins, however, high activation energies are applied for detergent removal. Therefore, generating ions with lower charge states may be advantageous as this could help maintain oligomeric state and prevent unwanted collision-induced dissociation of subunits.

**Figure 3:**
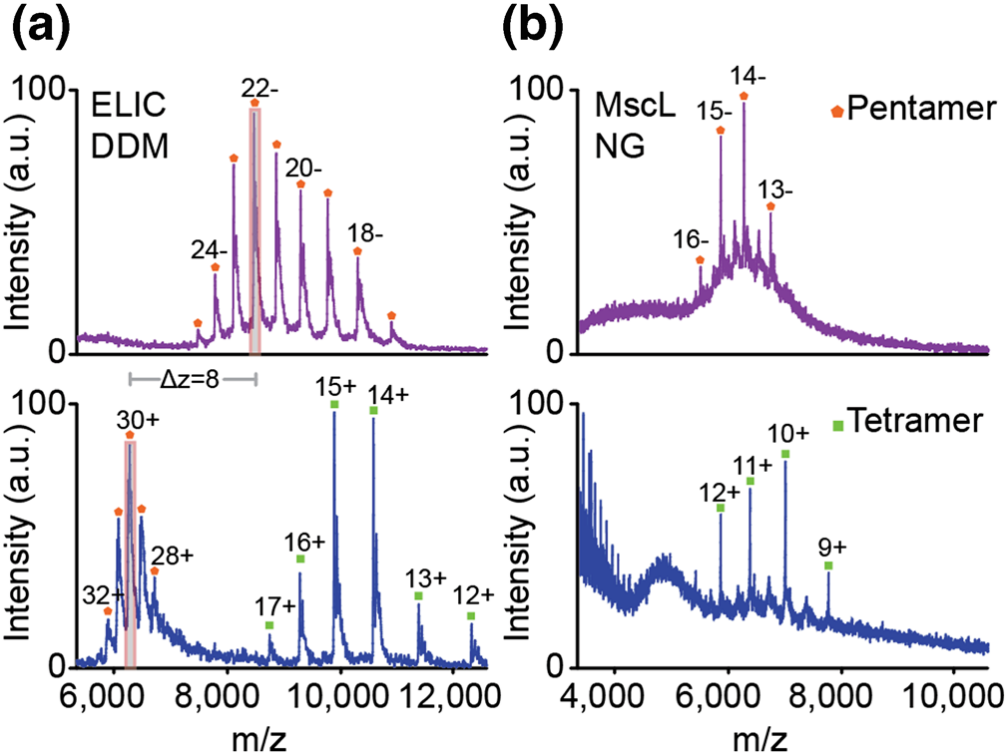
Charge reduction in negative polarity stabilizes the native oligomeric state. (**a**) nESI mass spectra of ELIC in DDM in both negative (top, purple) and positive (bottom, blue) polarities. The highest intensity charge states in both polarities are shaded grey. (**b**) Mass spectra of MscL solubilized in NG in negative polarity (top, purple) and in positive polarity (bottom, blue)

To investigate this possibility, mass spectra were recorded of two pentameric membrane proteins: *Erwinia chrysanthemi* ligand-gated ion channel (ELIC) and the *Mycobacterium tuberculosis* mechanosensitive channel of large conductance (MscL), solubilized in DDM and nonyl glucoside (NG) detergents, respectively (Figure 3). Both DDM and NG require higher activation energy for removal compared to C8E4, and are associated with higher charge states in the positive ion mode. Consequently, the native oligomeric states of ELIC in DDM and MscL in NG were not observed without some subunit dissociation. We attribute this difference to the energies required to disrupt the detergent and resolve the proteins. Both proteins then dissociate via collision induced dissociation (CID) into tetramer and monomer although some pentamer remains in the case of ELIC.

In contrast, when spectra were recorded for these proteins, using the same experimental conditions, in negative polarity, the native oligomeric state was preserved without generation of CID products in both cases. The negative ion spectrum of ELIC yields a charge-state shift of seven charges, solely from the change in instrument polarity. This shift in charge state was sufficient to completely eliminate subunit dissociation; the native pentameric state is the only oligomeric form observed (Figure 3). Therefore, in these two cases, negative polarity can be used to preserve the native oligomeric states of membrane proteins that are solubilized in saccharide detergents.

Spectra of MscL illustrate another advantage of using negative polarity to maintain oligomeric state. The energy required to remove the NG micelle is substantially higher than the maximum energy that can be tolerated while preserving the oligomeric state. As such, due to incomplete micelle removal, the pentameric oligomeric state cannot be resolved in the spectrum (Figure 3), and only CID products are apparent; the tetramer is well-resolved, whereas the monomer intensity cannot be easily visualized because of the presence of the detergent micelle in the low *m/z* region. Switching the polarity to negative preserved the pentameric species with the charge state distribution centred at 14.3–. Notably, this suggests that in the negative ion mode, for some detergents, less collisional activation is required for their removal despite the lower average charge state compared to positive polarity. Not only does this help preserve native oligomeric state, it will also help to maintain other noncovalent interactions of interest, such as those of small molecules, including lipids, drugs, and cofactors. In addition, spectra in the negative ion mode frequently have less background interference from detergents, particularly in the lower *m/z* regions (Figure 3 and Supplementary Figure S1), simplifying spectra and enhancing the resolution of peaks assigned to membrane proteins.

### Additive Effects of Negative Polarity Combined with Charge Reducing Agents

**Figure 4:**
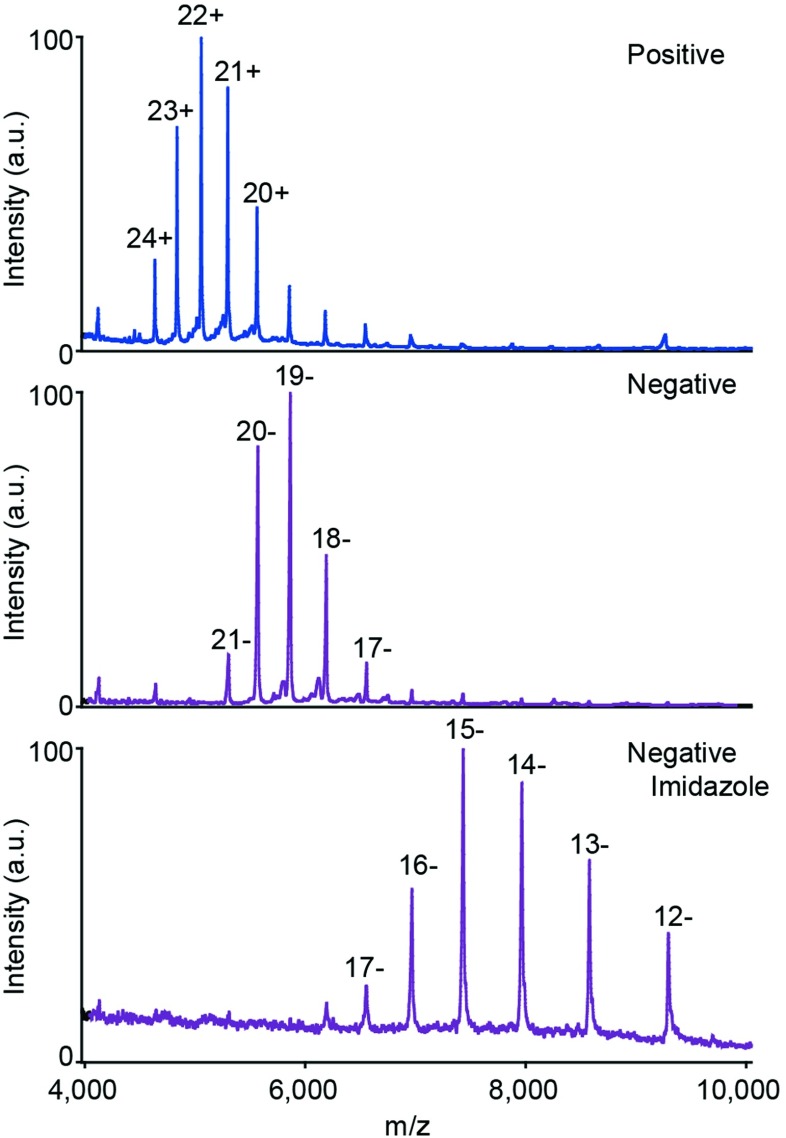
The charge reduction effects of negative polarity and charge reducing agents in solution can be combined to access lower charge states. (Top) Mass spectrum of OmpF in OG. (Middle) Negative polarity mass spectrum. (Bottom) Mass spectrum in negative polarity after addition of 5 mM imidazole to the OmpF containing solution

A common method to achieve lower charge states in positive polarity is the use of charge reducing agents, e.g., addition of millimolar concentrations of imidazole in the protein solution or exposure to acetonitrile vapors near the source region of the mass spectrometer [18–20]. Applying these approaches to membrane proteins in the negative ion mode may, therefore, allow access to even lower charges. To investigate this, we first recorded mass spectra of OmpF in positive mode. We observed an average charge state, with the protein solubilized in OG, of 21.9+. When a spectrum is recorded in negative polarity, the average charge state is reduced to 19.2–, approximately three charges lower than in positive mode (Figure 4). Surprisingly, adding imidazole to the OmpF-containing solution and recording a spectrum in negative polarity shows an additional reduction by four charges, bringing the average charge state to 14.6–. In addition to being amphoteric, the charge reducing effect in both positive and negative polarity of imidazole can be possibly attributed to evaporative cooling [20], which may result in smaller droplets and, hence, a lower overall charge. Negative polarity experiments combined with charge reducing agents, therefore offer the possibility to access super-charge-reduced states of membrane proteins. We conclude that the effects of the change in the polarity together with the charge reducing agent in solution, enable concomitant benefits in terms of preservation of non-covalent interactions.

An alternative approach to access lower charge states for soluble proteins is exposure of the electrospray plume to acetonitrile vapors [18]. We investigated if the same charge reduction is observed in negative polarity for membrane proteins. The mass spectra of OmpF in OG centered at 19.2– did not shift significantly after exposure to acetonitrile (Supplementary Figure S3). Combining both charge reducing agents also did not result in a further reduction of charge states. In this case, a slight increase in the average charge state was observed for OmpF solubilized in OG and supplemented with 5 mM imidazole increased from 14.6– to 15.9– after exposure to acetonitrile vapors. These results provide additional evidence that the charge modulation of imidazole and acetonitrile is provided via different mechanisms.

## Conclusion

In this study, we have shown that membrane proteins experience similar charge reduction effects to soluble proteins when analyzed under negative polarity ESI. Detergents can influence this process, with saccharide detergents promoting the largest charge reduction effects, and polyethylene glycol detergents preventing further charge reduction. The sensitivity towards detergents is further amplified by considering the difference in charge reduction observed for OG relative to DDM. Both detergents are similar chemically and will likely have similar proton affinity values.

Although no immediate advantages to using negative mode ESI for soluble proteins exist [12], we have highlighted some key advantages in the case of membrane proteins without compromise in sensitivity. Owing to the higher energy regimes necessary for membrane protein analysis compared with soluble proteins, reductions in charge state, coupled to the apparent volatile nature of some detergents, combine to produce gas-phase ions that exhibit more folded conformations and protect native oligomeric states. In a broader context, negative polarity has already been shown to provide some advantages for lipidomics, glycomics, and peptide analysis [21, 22]. The results shown here highlight the usefulness of negative polarity for studying membrane proteins, not only preserving oligomeric state but also providing enhanced quality of spectra with reduced adduct formation and detergent interference, key parameters for the observation of drug and lipid binding, the current challenges in the field.

## Electronic supplementary material

Below is the link to the electronic supplementary material.

ESM 1 (PDF 206 kb)

## References

[1] Marcoux J, Robinson CV (2013). Twenty years of gas phase structural biology. Structure.

[2] Barrera NP, Di Bartolo N, Booth PJ, Robinson CV (2008). Micelles protect membrane complexes from solution to vacuum. Science.

[3] Laganowsky A, Reading E, Hopper JT, Robinson CV (2013). Mass spectrometry of intact membrane protein complexes. Nat. Protoc..

[4] Clemmer DE, Hudgins RR, Jarrold MF (1995). Naked protein conformations: cytochrome *c* in the gas phase. J. Am. Chem. Soc..

[5] Breuker K, McLafferty FW (2008). Stepwise evolution of protein native structure with electrospray into the gas phase, 10–12 to 102 s. Proc. Natl. Acad. Sci. U. S. A..

[6] Reading E, Liko I, Allison TM, Benesch JLP, Laganowsky A, Robinson CV (2015). The role of the detergent micelle in preserving the structure of membrane proteins in the gas phase. Angew. Chem. Int. Ed. Engl..

[7] Privé GG (2007). Detergents for the stabilization and crystallization of membrane proteins. Methods.

[8] Mehmood, S., Marcoux, J., Hopper, J.T.S., Allison, T.M., Liko, I., Borysik, A.J., Robinson, C.V.: Charge reduction stabilizes intact membrane protein complexes for mass spectrometry. J. Am. Chem. Soc. **136**, 17010–17012 (2014) 10.1021/ja510283g PMC4594752 25402655

[9] Heck AJ, van den Heuvel RH (2004). Investigation of intact protein complexes by mass spectrometry. Mass Spectrom. Rev..

[10] Konermann L, Douglas D (1998). Unfolding of proteins monitored by electrospray ionization mass spectrometry: a comparison of positive and negative ion modes. J. Am. Soc. Mass Spectrom..

[11] Wyttenbach T, Grabenauer M, Thalassinos K, Scrivens JH, Bowers MT (2009). The effect of calcium ions and peptide ligands on the relative stabilities of the calmodulin dumbbell and compact structures. J. Phys. Chem. B.

[12] Allen SJ, Schwartz AM, Bush MF (2013). Effects of polarity on the structures and charge states of native-like proteins and protein complexes in the gas phase. Anal. Chem..

[13] Hogan CJ, Carroll JA, Rohrs HW, Biswas P, Gross ML (2009). Combined charged residue-field emission model of macromolecular electrospray ionization. Anal. Chem..

[14] Housden, N.G., Wojdyla, J.A., Korczynska, J., Grishkovskaya, I., Kirkpatrick, N., Brzozowski, A.M., Kleanthous, C.: Directed epitope delivery across the Escherichia coli outer membrane through the porin OmpF. Proc. Natl. Acad. Sci. U.S.A. **107**, 21412–21417 (2010) 10.1073/pnas.1010780107 PMC3003033 21098297

[15] Koppel DA, Kinnally KW, Masters P, Forte M, Blachly-Dyson E, Mannella CA (1998). Bacterial expression and characterization of the mitochondrial outer membrane channel effects of n-terminal modifications. J. Biol. Chem..

[16] Hernandez H, Robinson CV (2007). Determining the stoichiometry and interactions of macromolecular assemblies from mass spectrometry. Nat. Protoc..

[17] Allison, T.M., Reading, E., Liko, I., Baldwin, A.J., Laganowsky, A., Robinson, C.V.: Quantifying the stabilizing effects of protein-ligand interactions in the gas phase. Nat. Commun. **6**, (2015) 10.1038/ncomms9551 PMC4600733 26440106

[18] Hopper JTS, Sokratous K, Oldham NJ (2012). Charge state and adduct reduction in electrospray ionization-mass spectrometry using solvent vapor exposure. Anal. Biochem..

[19] Scalf M, Westphall MS, Krause J, Kaufman SL, Smith LM (1999). Controlling charge states of large ions. Science.

[20] Bagal D, Kitova EN, Liu L, El-Hawiet A, Schnier PD, Klassen JS (2009). Gas phase stabilization of noncovalent protein complexes formed by electrospray ionization. Anal. Chem..

[21] Schuhmann K, Almeida R, Baumert M, Herzog R, Bornstein SR, Shevchenko A (2012). Shotgun lipidomics on a LTQ Orbitrap mass spectrometer by successive switching between acquisition polarity modes. J. Mass Spectrom..

[22] Zaia J (2008). Mass spectrometry and the emerging field of glycomics. Chem. Biol..

