# Supplementary material for: Negative Ions Enhance Survival of Membrane Protein Complexes
Source: J Am Soc Mass Spectrom. 2016 Apr 22;27:1099–104. doi: 10.1007/s13361-016-1381-5 (PMC4869745; doi:10.1007/s13361-016-1381-5)
Supplement: Supplementary file 1 — (PDF 206 kb) [file 13361_2016_1381_MOESM1_ESM.pdf]

Electronic Supplementary Material

Negative ions enhance survival of membrane protein complexes

Journal of The American Society for Mass Spectrometry

Idlir Liko, Jonathan T.S. Hopper, Timothy M. Allison, Justin L.P. Benesch, Carol V. Robinson

Department of Chemistry, University of Oxford, South Parks Road, Oxford OX1 5QY, UK
carol.robinson@chem.ox.ac.uk

Table 1. Average charge states for selected proteins in both positive and negative polarity as dependent on the detergent type.

Proteins	Mass(kDa)	Average charge in positive polarity				Average charge in negative polarity			
		DDM	OG	C8E4	LDAO	DDM	OG	C8E4	LDAO
VDAC	31		9.1	6.4			7.2	7.1	
MATE	50	13.6		9.0		9.8		10.0	
AqpZ	99	20.2	18.1		14.6	14.5	15.1		16.2
OmpF	111		21.9	15.8			19.2	15.9	
*Ec*AmtB	127		21.4	16.3			18.1	17.4	
*Mt*AmtB	143			18.8				19.5	
ELIC	184	30.1				22.9			

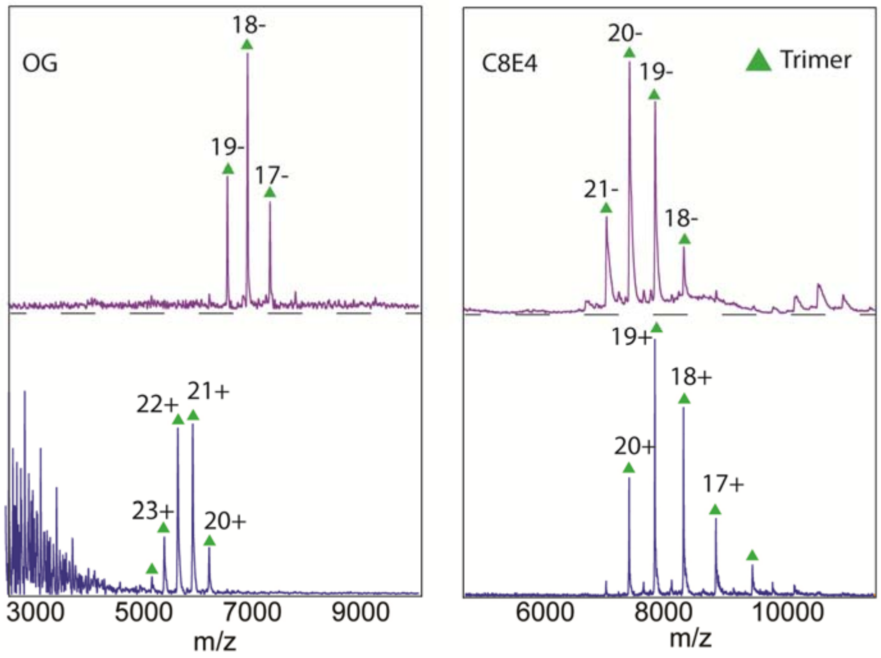

Figure S1: *n*ESI mass spectra of *Ec*AmtB and *Mt*AmtB solubilised in OG (left) and C8E4 (right) respectively. Measurements performed in positive polarity (blue spectra) and negative polarity (purple spectra) showed a charge reduction in OG and slight charge increase in C8E4.

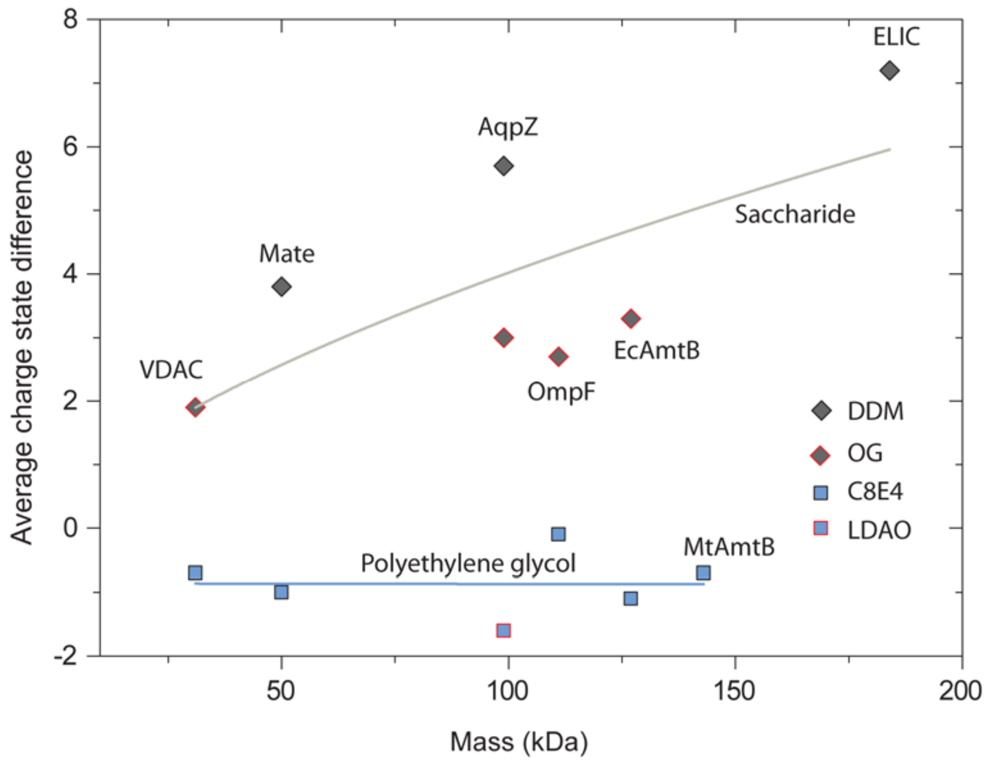

Figure S2: The effect of polarity on the average charge states in the presence of saccharide detergents and polyethylene glycol. The difference in average charge state of a range of membrane proteins as a function of protein mass between the two polarities when solubilised in saccharide detergents DDM or OG (grey) in C8E4 (blue) and LDAO (red square outline).

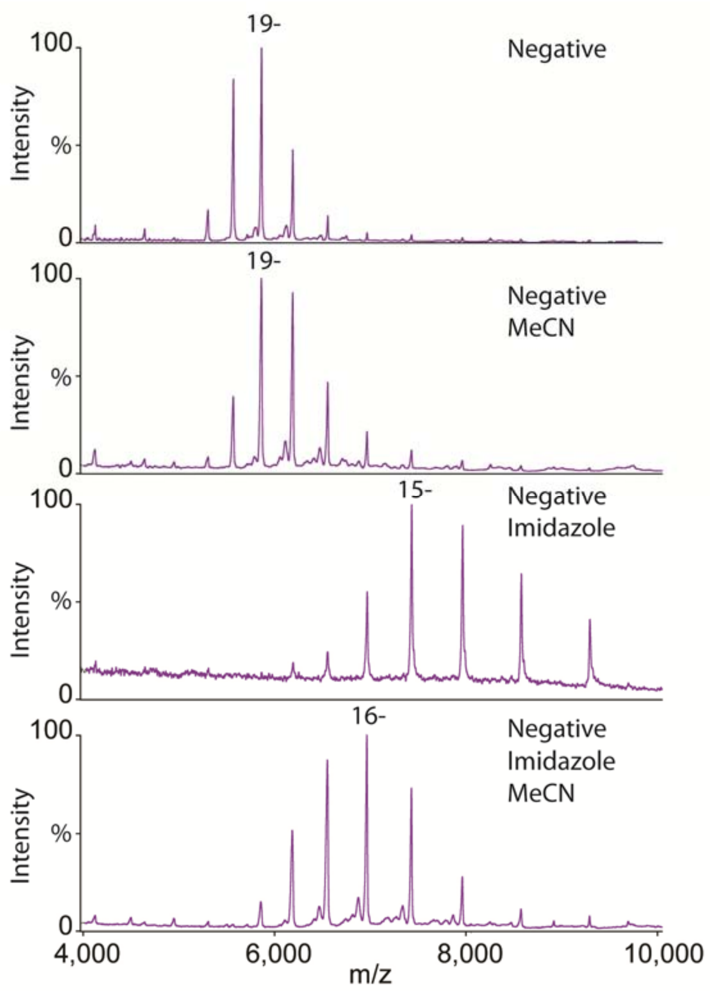

Figure S3: Mass spectra of OmpF solubilised on OG showing the effect of charge modulating agents in negative polarity. Relatively no change in charge state distribution is observed in the absence or presence of acetonitrile (MeCN) vapours (top two spectra). When 5mM imidazole is supplemented in the sample the exposure of MeCN vapours leads to a slight charge increase.
